# A modified Delphi method toward multidisciplinary consensus on functional convalescence recommendations after abdominal surgery

**DOI:** 10.1007/s00464-016-4931-9

**Published:** 2016-05-02

**Authors:** Daphne C. R. van Vliet, Eva van der Meij, Esther V. A. Bouwsma, Antonie Vonk Noordegraaf, Baukje van den Heuvel, Wilhelmus J. H. J. Meijerink, W. Marchien van Baal, Judith A. F. Huirne, Johannes R. Anema

**Affiliations:** 1 Department of Obstetrics and Gynaecology, VU University Medical Center, De Boelelaan 1117, 1081 HV Amsterdam, The Netherlands; 2 EMGO Institute for Health and Care Research, Amsterdam, The Netherlands; 3 Department of Obstetrics and Gynaecology, Flevoziekenhuis, Almere, The Netherlands; 4 Department of Surgery, VU University Medical Center, Amsterdam, The Netherlands; 5 Department of Public and Occupational Health, VU University Medical Center, Van der Boechorststraat 7, 1081 BT Amsterdam, The Netherlands; 6 Department of General Practice, VU University Medical Center, Amsterdam, The Netherlands

**Keywords:** Convalescence recommendations, Appendectomy, Cholecystectomy, Hernia repair, Colectomy, Modified Delphi study

## Abstract

**Background:**

Evidence-based information on the resumption of daily activities following uncomplicated abdominal surgery is scarce and not yet standardized in medical guidelines. As a consequence, convalescence recommendations are generally not provided after surgery, leading to patients’ insecurity, needlessly delayed recovery and prolonged sick leave. The aim of this study was to generate consensus-based multidisciplinary convalescence recommendations, including advice on return to work, applicable for both patients and physicians.

**Method:**

Using a modified Delphi method among a multidisciplinary panel of 13 experts consisting of surgeons, occupational physicians and general practitioners, detailed recommendations were developed for graded resumption of 34 activities after uncomplicated laparoscopic cholecystectomy, laparoscopic and open appendectomy, laparoscopic and open colectomy and laparoscopic and open inguinal hernia repair. A sample of occupational physicians, general practitioners and surgeons assessed the recommendations on feasibility in daily practice. The response of this group of care providers was discussed with the experts in the final Delphi questionnaire round.

**Results:**

Out of initially 56 activities, the expert panel selected 34 relevant activities for which convalescence recommendations were developed. After four Delphi rounds, consensus was reached for all of the 34 activities for all the surgical procedures. A sample of occupational physicians, general practitioners and surgeons regarded the recommendations as feasible in daily practice.

**Conclusion:**

Multidisciplinary convalescence recommendations regarding uncomplicated laparoscopic cholecystectomy, appendectomy (laparoscopic, open), colectomy (laparoscopic, open) and inguinal hernia repair (laparoscopic, open) were developed by a modified Delphi procedure. Further research is required to evaluate whether these recommendations are realistic and effective in daily practice.

In the last decade, enhanced recovery after surgery (ERAS) or fast track programs to speed up discharge after surgery have become increasingly popular [1–3]. This, together with the introduction of minimally invasive surgery, causes more surgical procedures to be performed in day- or short-stay care, leading to an early transfer of the postoperative care to the primary healthcare professionals. However, hardly any attention so far has been focused on the rehabilitation following hospitalization; evidence-based information on when and how to gradually resume daily activities including work after uncomplicated surgery is scarce, and uniform multidisciplinary recovery recommendations are not yet standardized in medical guidelines [4–6].

Due to the limited evidence on recovery advice, the majority of caregivers involved in this process provide patients with experience-based recommendations [4, 7–10]. Different groups of healthcare professionals are exposed to diverse patients after diverse kinds of surgery, resulting in a wide variety of opinions on convalescence abilities after that particular surgery. For example, postoperative follow-up by the operating surgeon will be executed in an early stage after surgery, whereas occupational physicians (OPs) will be consulted relatively late in the course of sick leave by patients with a delayed recovery only. General practitioners are seldom consulted by patients on the resumption of activities or work [11, 12].

As a consequence, patients often receive conflicting advice from involved care providers leading to insecurity on when to resume various activities after surgery [13]. In addition, compliance to these diverse recommendations is difficult and therefore low [4, 9, 14, 15]. This may contribute to a delayed recovery and prolonged sick leave [11]. Subsequently, prolonged absence from work and return to daily activities may result in a poorer emotional well-being and have major socioeconomic consequences [14, 16].

The literature shows that duration of time to return to work (RTW) is influenced by patients’ expectations on time to return to work [17]. Studies investigating the influence of postoperative advice on when to resume activities and work [7, 18, 19] state that uniform convalescence recommendations have a positive effect on early resumption of daily activities and work [20].

This underlines the need for accurate information on when to resume various activities and work following surgery. To improve recommendations on patients’ expectations and to provide a guiding tool for physicians, the development of multidisciplinary convalescence consensus is essential.

The aim of this modified Delphi study is to develop uniform, multidisciplinary convalescence recommendations, designed for the most frequently performed general abdominal surgical interventions in the Netherlands: laparoscopic cholecystectomy, laparoscopic and open appendectomy, laparoscopic and open inguinal hernia repair and laparoscopic and open colectomy.

## Materials and methods

### Design of a modified Delphi study

The Delphi technique is a method with the aim to develop a consensus opinion on a specific subject within an expert group in a structured way [21]. Through repeated anonymous questionnaire rounds, the experts are provided with the opportunity to reflect on the results of the previous questionnaire round in a controlled manner. A Delphi procedure is successfully completed as soon as consensus is reached according to a previously defined consensus rule, or when the investigator concludes that consensus is not increasing in following rounds: in other words, when it turns out that experts are not prepared to alter their point of view anymore.

**Fig. 1:**
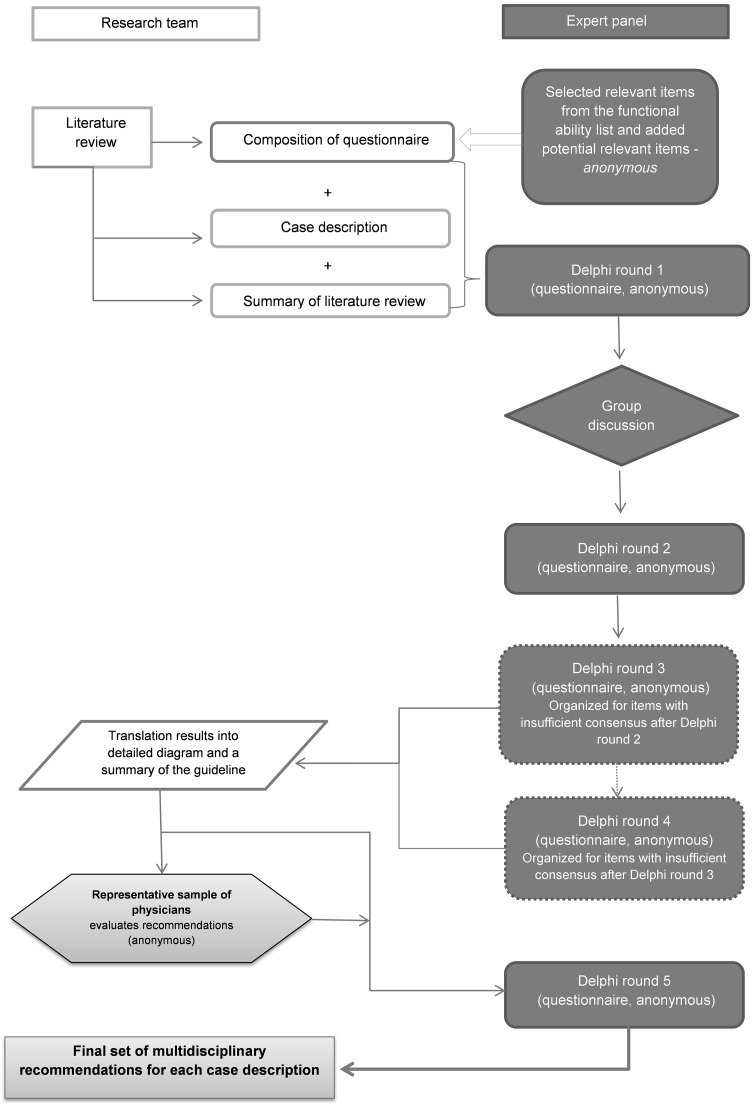
Study design; the stepwise modified Delphi method used in this study to reach a multidisciplinary consensus on convalescence recommendations

The Department of Gynecology and Obstetrics, the Department of Public and Occupational Health and the Department of General Practice of the VU Medical Centre have demonstrated that a modified Delphi procedure is a useful tool in achieving consensus on when to resume work and daily activities after uncomplicated abdominal gynecological surgery [22]. In the present Delphi study, we have used a similar design to achieve consensus on various general surgical procedures: laparoscopic cholecystectomy, laparoscopic and open appendectomy, laparoscopic and open inguinal hernia repair and laparoscopic and open colectomy. In addition to the structured repeated anonymous questionnaire rounds, one live panel discussion meeting was organized. For the group discussion, a nominal group technique was used to reach consensus [23]. The data were collected between February and November 2014. The study design is presented in Fig. 1.

### Literature review

A review of the literature in five international databases (PubMed, EMBASE, The Cochrane Library, CINAHL and PsycInfo) published until 2013 was performed with the assistance of a medical information specialist. Searches were carried out for convalescence recommendations and time to return to normal activities (RNA) and time to return to work (RTW) as primary outcome measures after cholecystectomy, appendectomy, colectomy and inguinal hernia repair. Search terms used included the following mesh terms as well as a combination of free text words and mesh terms in title or abstract: “Colectomy,” “Appendectomy,” “Cholecystectomy,” “Herniorrhaphy,” “Hernia repair,” “Absenteeism,” “Convalescence,” “Recovery of Function,” “Sick Leave,” “Disability Evaluation,” “Work Capacity Evaluation,” “Rehabilitation,” “Vocational,” “Return to Work” and “Sickness Impact Profile.”

Papers were assessed for eligibility by two researchers (EB, DVV) by a list of predefined inclusion criteria. Only studies reporting RNA or RTW as their primary or secondary outcome were included. Study types other than randomized controlled trials (RCTs), systematic reviews or international guidelines were excluded. During the process, it was decided to only select studies from 1990 onward because of the large number of eligible studies. All recovery times and recommendations reported in the included papers were summarized, and this review of the literature was provided to all expert panel members to be used as a guidance while completing the first Delphi questionnaire round.

### Case definition and draft case description

For each surgical intervention, a case description was designed to be used by the expert members as a reference point while completing the questionnaires. These case descriptions outlined an uncomplicated surgical procedure in otherwise healthy patients without any comorbidity.

### Development of a list with relevant convalescence recommendations

The Functional Ability List (FAL) was used to develop convalescence recommendations. This instrument distinguishes 59 different physical and psychosocial activities (e.g., lifting and concentrating) and provides an overview of an individual’s general functional abilities. In the Netherlands, it is used by OPs and insurance physicians (IPs) to assess and advise patients in their functional abilities in daily life and at work.

Experts were asked to determine which of the 59 items of the FAL were considered relevant in the recovery of patients in the perspective of the surgeries described and were able to propose additional activities to design recovery recommendations for.

### Consensus rules

A set of consensus rules was used to identify on which FAL item the experts consented and which FAL items did not yet reach consensus. In case no consensus was reached, the particular FAL item had to be scored again by the experts in the following questionnaire round. Consensus for dichotomous items was reached when consensus at all individual time points was at least 75 %. For items with three or more grades of ability, consensus was reached when consensus over all time points exceeded 66.7 %.

### Expert panel recruitment

During the formation of the expert panel, it was important to select members that resemble the different types of caregivers that are involved in the guidance of patients recovering from surgery, as they all have their own focus during the recovery period. The members of the expert panel, consisting of seven surgeons, three occupational physicians (Ops) and three general practitioners (GPs), were recruited from different hospitals and professional organizations/boards in the Netherlands. Surgeons, all practicing minimally invasive surgery according to modern care standards, were recruited at different district hospitals as well as academic centers in the Netherlands, taking into consideration each individual expertise on the investigated surgical procedures. GPs were recruited using the network of an academic center for the training of family practice. None of the members of the expert panel reported to have potential conflicts of interest.

### Description of the structural consensus method

#### Delphi questionnaire rounds and group meeting

**Fig. 2:**
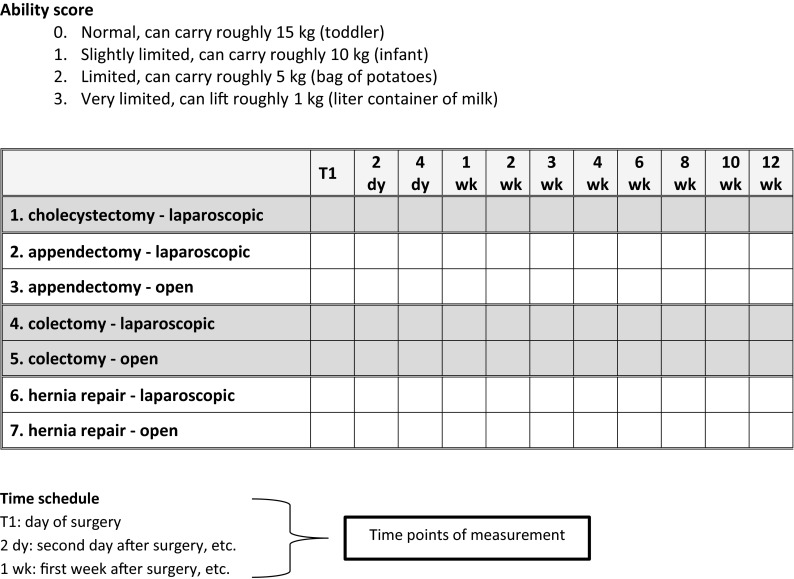
Example of the item carrying and lifting of the functional ability list

In the first round, the functional ability of each activity (FAL item and additional activities) was scored on the day of surgery and at 11 different time points following surgery by each of the panel members individually for all seven case descriptions (laparoscopic cholecystectomy and laparoscopic as well as open appendectomy, colectomy and inguinal hernia repair). In this way, the gradual resumption of the activity could be visualized. For example, it was asked when patients were expected to be able to carry 2, 5, 10 and 15 kg (see Fig. 2).

The mode and median values of the ability scores for each item anonymously obtained in the first Delphi round were graphically presented to the experts in a group meeting. During this group meeting, it was possible to explore the items in which a wide variance of opinions were identified and the meeting provided the experts with the opportunity to gain insight in the reasons for the wide variation according to their frame of reference concerning this topic. After the group discussion, all experts were asked to anonymously rate the ability score for the specific items again (Delphi round 2), taking into consideration that the most restrictive ability score had to be chosen in the event of uncertainty.

After this round, the following questionnaire round asked to rate the functional ability score once more for the items at the individual time points where consensus had not yet been reached, taking into consideration the most frequently chosen ability score (mode) at this specific time point calculated in the second Delphi round.

In the subsequent questionnaire round, the results of the prior round were presented to the experts. For those items that did not yet reach consensus, we asked the experts again to anonymously reflect their opinion. In addition to the median and the mode scores, we also provided the experts with the following details to help them choose the score that fitted best, taking into consideration the consented ability scores on other time frames of the same procedure and on the same time point for other surgical procedures:

- The ability scores of all other FAL items for that same surgical procedure at that particular time point the experts consented on;
- The ability scores of that specific FAL item on that specific time point in relation to the other surgical procedures;
- The consensus opinion on the similar FAL item on that specific time point for adnexal surgery and hysterectomy, conducted in our previous Delphi study.

#### Evaluation of the feasibility of recommendations by a sample of physicians

A detailed overview of the consensus reached by the expert panel members after the first four Delphi rounds was sent to representatives of the same professional groups as the expert panel members. A total of 40 representatives were asked to participate. Of these, 18 physicians were able to do so. These 18 representatives, consisting of six OPs, seven GPs and five surgeons, judged the feasibility of the recommendations in daily practice.

#### Final Delphi questionnaire round

The consensus opinion reached after the Delphi questionnaire rounds and one group meeting was schematically presented to the expert panel in the final Delphi questionnaire round, together with the feasibility judgment of the sample of physicians. The experts could reflect on the comments of the sample of physicians and if necessary reconsider their opinion.

## Results

### Review of the literature

The literature search resulted in 2454 papers. All titles and abstracts were reviewed, and cross-references of relevant papers were checked. A total of 65 papers seemed potentially relevant. After assessing the eligibility, six full-text articles [7, 24–28] were sent to all panel members accompanied by a summary of the reported results of 35 papers and one international guideline existing of: nine RCTs and one prospective study for cholecystectomy (regarding RTW [29–34], regarding RNA [29, 30, 33]), 13 RCTS for appendectomy (six regarding RTW [27, 35–39] and seven regarding RNA [35, 36, 38, 40–43]), six studies for colectomy (regarding three on RTW described in one review study [27] and two on RNA [44, 45]) and two systematic reviews [25, 46], one international guideline [47] and two prospective studies on hernia repair [48]. None of these studies reported gradual resumption of various activities after surgery, but most reported on general “return to leisure or daily activities,” without underlying definitions.

### List of relevant convalescence recommendations

Out of 56 activities of the FAL, the expert panel selected 26 relevant activities to develop convalescence recommendations for. The 26 FAL items were included in the Delphi procedure, together with five additional activities (*taking a bath, jumping, vacuum cleaning and sexual intercourse* (*men and women*)). During the group meeting, the experts decided that in the second Delphi questionnaire round, two additional activities of importance in the recovery of patients should be added: *riding a bike* and *driving a car*. In Delphi round 3, the experts asked to add item *public* *transportation*. Also, the experts agreed on the fact that FAL item *concentrating* is influenced by the form of anesthetics that is used, irrespective of the type of surgery the patient is undergoing. Therefore, this item was divided into regional or local anesthetics and scored at the different time points in Delphi rounds 3 and 4. A total of 26 FAL items and eight additional activities, meaning 34 activities all together, were evaluated.

### Expert panel

The expert panel consisted of seven surgeons, all performing minimally invasive surgery [two women (32 and 53 years) and five men (range 37–52 years)], three general practitioners [one man (47 year) and two women (34 and 37 years)] and three occupational physicians [three men (range 51–57 years)]. All of them had the Dutch nationality.

### Consensus course

#### Number of Delphi rounds and response rate

Five questionnaire rounds and one expert group meeting were required to meet the objectives of the study. The response rate for all rounds was 100 %. All experts completed the entire study.

#### First Delphi questionnaire round

After the first Delphi questionnaire round, the consensus per time point and the mean consensus were calculated for each item. Regarding all surgical procedures, there were no items that reached overall consensus, meaning no consensus at every individual time point was reached.

#### Delphi questionnaire rounds 2, 3 and 4

Table 1 illustrates the flow of minimal consensus reached per individual time point for laparoscopic cholecystectomy. As shown in this table, for cholecystectomy in round 2, nine out of 31 items met the previously defined criteria for the consensus rule. In Delphi round 3, consensus was reached for 17 out of the 34 items. After the fourth Delphi questionnaire round, consensus on all 34 activities was reached. The other surgical procedures were judged in a similar manner, and for each of the 34 items regarding all seven surgical interventions, consensus was reached.

**Table 1:** Course of minimum consensus reached per individual time point for cholecystectomy

FAL item	Max. number of existing gradations in which item is expressed	Round 1 (%)	Round 2 (%)	Round 3 (%)	Round 4 (%)
Reaching out	3	46.2	*61.5*	*61.5*	**100.0**
Reaching out frequently	4	46.2	*61.5*	**84.6**	**100.0**
Bending	3	*38.5*	*61.5*	**76.9**	**100.0**
Bend frequently	4	*53.8*	**69.2**	**100.0**	**100.0**
Turning/twisting round	2	*46.2*	*53.8*	*61.5*	**100.0**
Pushing/pulling	3	*46.2*	*61.5*	**84.6**	**100.0**
Lifting or carrying	4	38.5	**76.9**	**100.0**	**100.0**
Handle light objects frequently	4	30.8	*46.2*	*61.5*	**100.0**
Handle heavy objects frequently	2	*69.2*	*69.2*	*69.2*	**92.3**
Sustained Walking	4	46.2	**69.2**	**100.0**	**100.0**
Walking per day	4	*53.8*	*61.5*	**84.6**	**100.0**
Climbing stairs	4	*50.0*	**100.0**	**100.0**	**100.0**
Climbing	4	41.7	*46.2*	*53.8*	**92.3**
Kneeling or squatting	2	*63.6*	**76.9**	**100.0**	**100.0**
Prolonged sitting	4	41.7	*61.5*	**76.9**	**100.0**
Sitting per day	4	41.7	*53.8*	**92.3**	**100.0**
Prolonged standing	4	*58.3*	*46.2*	*53.8*	**84.6**
Standing per day	4	46.2	*53.8*	**84.6**	**100.0**
Actively kneeling	2	*61.5*	**84.6**	**100.0**	**100.0**
Actively bending	2	*53.8*	*53.8*	*61.5*	**84.6**
Working above shoulders	2	*61.5*	*69.2*	*69.2*	**100.0**
Working hours per day	5	38.5	*38.5*	*38.5*	**100.0**
Working hours per week	5	30.8	*46.2*	*46.2*	**100.0**
Working hours shift work	3	30.8	41.7	*46.2*	**92.3**
Taking a bath^a^	2	*53.8*	*61.5*	*61.5*	**92.3**
Jumping^a^	2	*61.5*	*61.5*	*53.8*	**92.3**
Vacuum cleaning^a^	2	53.8	*69.2*	**92.3**	**100.0**
Driving a car^b^	2	–	*61.5*	**76.9**	**100.0**
Riding a bicycle^b^	2	–	*53.8*	*61.5*	**100.0**
Sexual intercourse (man)^a^	2	53.8	*61.5*	*53.8*	**76.9**
Sexual intercourse (woman)^a^	2	53.8	**76.9**	**100.0**	**100.0**
Concentrating	3	53.8	**84.6**	–	–
Insight into own abilities	3	*66.7*	**100.0**	**100.0**	**100.0**
Transportation^c^	2	–	–	*61.5*	**92.3**
Concentrating (2)*	3	–	–	50.0	**100.0**

Italic: Mean consensus reached, but contains individual time point with consensus <66, 7 % for categorical or <75 % for dichotomous parameters

Bold: Consensus reached at every individual time point

Endash: Particular FAL item was not questioned this round

* This item the experts judged to be influenced by the type of sedation given (regional or local anesthetics); therefore, this item was adjusted from round 3 onward

^a^Additional item

^b^Additional item after first Delphi round

^c^Additional item after second Delphi round

#### Evaluation of the feasibility of recommendations by a representative sample of physicians

For all procedures, the 18 physicians of the sample judged the consensus as feasible in daily practice. Only minor revisions were requested.

#### Fifth Delphi round

In this round, the experts reflected on the comments of the sample of physicians. The few minor revisions the sample requested were judged as irrelevant by all 13 experts. Therefore, no adjustments were made to the draft recommendations.

### Final convalescence recommendations and case descriptions

A final set of convalescence recommendations was formulated for each case description, based on the consensus findings after Delphi round 4 and comments of the sample of physicians. Table 2 illustrates how the recommendations may be summarized as guidelines for all surgical procedures.

**Table 2:** Summary of the final set of multidisciplinary recommendations regarding abdominal surgery

Handling capacity From light to strenuous activity		Hernia repair laparoscopic	Hernia repair open	Cholecystectomy laparoscopic	Appendectomy laparoscopic	Appendectomy open	Colectomy laparoscopic**	Colectomy open**
↓	2-h sustained sitting 30-min sustained sitting or walking Climbing at least one staircase up and down Lifting or carrying 5 kg	2–4 dys	2–4 dys	2–4 dys	2–4 dys	4 dys	4 dys–1 wk	1–2 wks
	Sitting during entire day 4-h standing a day 4-h walking a day Lifting or carrying 15 kg	1 wk	1 wk	1–2 wks	1–2 wks	1–2 wks	3–4 wks	4 wks
	Standing and walking during entire working day	2 wks	2 wks	3 wks	2–3 wks	3 wks	4 wks	6 wks
	*Other activities* Driving a car Riding a bicycle Taking a bath Sexual intercourse man*** Sexual intercourse woman***	1 wk	1 wk	1 wk	1 wk	1 wk	2 wks 2 wks 1 wk 1 wk 1 wk	2 wks 3 wks 1 wk 1 wk 2 wks
	*Resumption of (average) job* ± 8 h a day ± 40 h a week	2 wks	2 wks 3 wks	2 wks 3 wks	2 wks 3 wks	2 wks 3 wks	6 wks	8 wks

The resumption of activities is considered medically safe from the presented days/weeks after day of surgery, although individual factors may account for variation in post-operative recovery

Wk(s), week(s); Dy(s), days

** In case of adjuvant chemo-/radiation therapy, additional advice is required

*** Considered medically safe, but may still be painful

## Discussion

### Main findings

The modified Delphi method proved to be an efficient and useful method in achieving multidisciplinary consensus on convalescence recommendations following uncomplicated abdominal surgery. Consensus was reached on 34 relevant activities after four questionnaire rounds and one group meeting. The recommendations were judged to be feasible for use in daily practice by a sample of physicians.

### Strengths and limitations

The strength of this study lies in its design: a modified Delphi method. Main advantages of this method are fourfold: First is the heterogeneity of the expert panel, resembling the different caregivers’ occupations involved in the guidance of patients in their postoperative recovery period, since they all have their own focus during this process. This answered our aim to reach multidisciplinary consensus. Second is the systematic collection of evidence concerning this topic, of which the experts received an overview. Third, the design of the study allowed experts to complete all questionnaires anonymously, preventing domination by any individual who might otherwise be overly influential. Fourth, the group meeting provided a setting in which reflection was possible and revision of earlier judgments of FAL items could take place. Furthermore, all experts completed the entire Delphi procedure without any dropouts.

A limitation of this study is the use of the functional ability list, which was originally developed for the detailed assessment of functional ability by OPs and IPs in the Netherlands. In our modified Delphi study, however, we used it to judge different gradations of strain in the recovery process after abdominal surgery. To date, there is no better suitable instrument available for the measurement and judgment of graded resumption of activities after surgery. Secondly, it could be questioned whether the sample of involved physicians is representative for all professionals involved, since the group of experts and the sample of physicians were not randomly selected and both groups consisted of a relatively small number of participants. The advantage of having a group with only 13 experts is that it is easier to discuss with each other and to hear everyone’s opinion based on daily practice and experiences. In addition, the level of evidence should stay the same, also if the group consisted of more professionals. We believe that 13 group members and 18 additional representatives should be enough to judge the recommendations. However, in order to evaluate whether these recommendations are realistic, future research with a bigger sample of patients and healthcare professionals is necessary. In addition, all physicians were from the Netherlands. Cultural differences could play a role in recovery and recovery recommendations, so external validity has to be examined for the results to be internationally applicable. It needs to be noted that formulated recommendations are only valid for healthy patients undergoing uncomplicated abdominal surgery and that in case of complications or comorbidities the physician will have to decide whether the convalescence recommendations need to be adapted.

No patients have participated in this Delphi study, which could be considered as a limitation of the study. We decided not to do so since in general patients underestimate their ability on RTW and RNA. Several factors play a role in this, and one of them is that the positive effects of minimally invasive approaches on recovery and RNA abilities are not known by patients [6, 8, 49, 50]. The greatest benefit of the development of uniform multidisciplinary recovery guidelines is the opportunity to manage patients’ expectations and cognitions regarding RTW and RNA. However, we did not neglect the importance of patient participation. The convalesce recommendations that are developed will be evaluated in an RCT, which will be described later in this discussion.

### Comparison with other studies

Uniform, multidisciplinary guidelines on when to resume daily activities and work after cholecystectomy, appendectomy, colectomy and inguinal hernia repair do not yet exist. The participation of all different healthcare specialists involved in a patient’s recovery process—from the moment surgery was scheduled until the return to daily and work-related activities—in the development of guidelines in these patients, is unique.

To our knowledge, there is one other study that used the Delphi technique to describe the resumption of six recovery-related activities after cholecystectomy, appendectomy and inguinal hernia repair (both open and laparoscopic) [51]. This Delphi study asked surgeons to consent on when it should be medically safe to resume the six activities that patients judged relevant in their recovery: “stretching,” “undertaking strenuous exercise,” “having sex,” “taking a bath,” “driving a car” and “being free of pain.” Comparing the recommendations of this study group to our own results demonstrates that the recommendations formulated by our own expert panel concerning the resumption of “strenuous activities” after laparoscopic and open inguinal hernia repair and appendectomy are less restrictive. Furthermore, our study is more extensive as our experts developed recommendations for the gradual resumption of 34 activities instead of considering only six single activities relevant. Finally, we consider our recommendations to be more representative for all stakeholders, as they were formulated by our multidisciplinary expert panel resembling all professionals of importance in the recovery period (surgeons, OPs and GPs) instead of only regarding surgeons’ opinion on the expected recovery.

In 2009, our department executed a similar modified Delphi study for the development of convalescence recommendations after gynecological surgery [22]. The modified Delphi method proved to be successful in bridging the gaps in opinions between the different stakeholders (in this case gynecologists, OPs and GPs) and to achieve consensus in a relatively short period of time. After four questionnaire rounds and two group meetings, consensus was reached for all relevant recommendations for resumption of activities after hysterectomy (vaginal, abdominal and laparoscopic) and after adnexal surgery (laparoscopic). Convalescence recommendations developed in both studies turned out to be similar for comparable procedures. For example, both expert panels agreed that it is medically safe to resume light activities after 2 days and to resume strenuous activities (standing and walking the entire day) after 2 weeks following both laparoscopic adnexal surgery and inguinal hernia repair (laparoscopic as well as open). Furthermore, recommendations following abdominal hysterectomy were quite comparable to the recommendations following open colectomy, with this difference that the resumption of an average job (8 h a day, 40 h a week) was considered medically possible after 8 weeks following open colectomy instead of the 6 weeks following an abdominal hysterectomy.

The convalescence recommendations that were developed in the gynecological Delphi study were evaluated in an RCT [20]. Patients who had access to the recommendation returned to work 9 days earlier than patients from the control group. Regarding the feasibility, it can be reported that in total 11 % (12/110) of the patients in the RCT stated that the recommendations were too conservative. On the other hand, 21 % (23/110) of these patients reported that the reintegration plan they had composed was too optimistic for their own situation. The majority of patients, 83 % (87/105), followed most convalescence recommendations. Since the recommendations that were evaluated in the present Delphi study turned out to be very similar, this suggests that the recommendations should also be realistic. However, also after this Delphi study future research is necessary to validate the recommendations.

Considering international guidelines on advice on RTW or RNA such as the UK guideline from the Royal College of Surgeons (RCS), the RCS developed patient leaflets that offer a broad guideline in recovery advice after uncomplicated surgery. These leaflets are accompanied by a “recovery tracker,” which globally describes how someone might feel after the specific surgery and offers some suggestions about what exercises to undertake postoperatively. This guideline recommends a postoperative recovery to full activity or work of 1–2 weeks after open inguinal hernia repair and of 2–3 weeks after laparoscopic cholecystectomy [52]. These recommendations are in line with those developed by our expert panel for a full return to physically demanding work, with the difference in that our recommendations take notion of the gradual resumption of activities. The clinical guidelines of the American Disability Advisor (MDA) present important time points at which additional evaluation should take place, if full recovery has not occurred; their disability guideline tables are designed to determine the duration of sickness benefit. In case of uncomplicated cholecystectomy, it states that most individuals should be able to resume normal activities within 7–10 days [53]. Upon return to work after inguinal hernia repair, individuals should not lift anything heavy for 6–8 weeks after surgery according to the MDA recommendations [54]. On the other hand, the European Hernia Society guidelines on the treatment of inguinal hernia in adult patients state that the imposition of a temporary ban on lifting, participating in sports or working after inguinal hernia surgery, is not necessary and as they quote “Probably a limitation on heavy weight lifting for 2–3 weeks is enough” [47]. The latter is in line with the recommendations of our expert panel.

Following uncomplicated appendectomy, most individuals are discharged from hospital within 1 day after surgery. The MDA states that activity will be limited for 1–3 weeks following surgery, but full recovery should be expected within 4–6 weeks and temporary restrictions on lifting are advised (not exceeding 11 kg for 6 weeks) [55]. Our Delphi panel judged that there is no need to restrict lifting or carrying of 15 kg after 1–2 weeks. For colectomy, a well-defined advice on when and how to convalesce is lacking, and according to the MDA, return to work and resumption of light activities should be approved by the surgeon [56].

Apart from the above-mentioned activities in the MDA guideline tables, an overview of the gradual resumption of recovery-related activities is not provided and therefore no timeline-related advice can be given for the resumption of specific activities in the recovery period of patients. The differences in recovery recommendations stated by the MDA compared with those of our Delphi expert panel are most plausibly explained through the fact that MDA guidelines are developed to determine the duration of sickness benefit from an insurance perspective; if full recovery does not occur in a certain timeframe, additional evaluation should take place. Our guideline, on the other hand, is developed to provide patients as well as doctors with accurate, uniform information about the expected time of recovery, reintegration and the gradual resumption of activities.

### Interpretation of the results and policy implications

The recommendations developed through our Delphi study can be interpreted as an average functional recovery time for the otherwise healthy adult patient. If complications or comorbidities are present, the physician will have to determine whether the recovery period needs to be extended. For example, concerning an appendectomy our expert panel decided the recommendations needed to be adjusted in case of a perforated appendectomy.

With the development of these multidisciplinary uniform convalescence recommendations, we aimed to provide surgeons, OPs and GPs with a tool to help them advise their patients at different moments in their recovery process. Convalescence is difficult to monitor. Unambiguous advice is of great importance to enhance recovery and social participation, including RTW [7, 57]. Convalescence recommendations given by healthcare providers, however, still show a great diversity [4, 9]. Patients often do not know whom to contact in case of questions or problems related to their recovery process. Standardized recommendations have become increasingly relevant since the introduction of ERAS or fast track programs and minimally invasive surgical techniques. Despite the related early transfer from hospital to primary care, hardly any attention so far has been focused on the rehabilitation after hospitalization. Well-defined postoperative instructions will likely have a positive effect on reducing sick leave and will motivate the patient to resume activities with increasing gradations of strain [7, 24, 58, 59].

Minimally invasive surgery potentially has major advantages over traditional surgery, not only from a patient’s (recovery) perspective but also on a socioeconomic scale. In order to take full benefit of these advantages, it is needed to optimize perioperative counseling and to develop multidisciplinary detailed recommendations on RNA and RTW after all types of surgery.

### Future perspectives

Now that multidisciplinary convalescence recommendations are developed, the next step will be to validate these recommendations within a sample of patients undergoing the particular types of surgical procedures.

From 2008 onward, our study group invested in optimizing perioperative care through the development of a multidisciplinary care program [13]. This care program consisted of an eHealth intervention providing guidance to patients undergoing benign gynecological surgery from the preoperative phase until full recovery of daily activities and work. Simultaneously, it evaluated the effectiveness of the convalescence recommendations of the 2009 Delphi study in clinical practice [60]. The multidisciplinary eHealth intervention proved to be an effective tool on reducing sick leave and improving quality of life and pain in patients after undergoing surgery [20]. These findings underline the need for uniform multidisciplinary convalescence advice after more types of surgery.

In line with this, the effectiveness of the recommendations of the present Delphi study needs to be evaluated in clinical practice through an RCT. Currently, we are designing this RCT in which the intervention group of patients will be equipped with tailored convalescence advice and integrated clinical and occupational care management for patients with prolonged sick leave is facilitated, compared to usual care. The primary outcome will be duration until resumption of daily activities. In addition, healthcare professionals from the participating hospitals will be asked to judge the recommendations.

## Conclusion

A multidisciplinary expert team consisting of surgeons, GPs and OPs, achieved consensus on convalescence recommendations regarding gradual resumption of daily activities and work after laparoscopic cholecystectomy and laparoscopic as well as open inguinal hernia repair, colectomy and appendectomy. At present, study toward validating the effectiveness of the recommendations in clinical practice is conducted.

In order to take full benefit of the potential advantages of minimally invasive surgery, it is recommended to optimize perioperative counseling and to develop multidisciplinary detailed recommendations on RNA and RTW after more types of surgery.
